# Oxidative Stress As A Common Mediator for Apoptosis Induced-Cardiac Damage in Diabetic Rats

**DOI:** 10.2174/1874192400802010070

**Published:** 2008-08-07

**Authors:** Mohammad M Dallak, Dimitri P Mikhailidis, Mohamed A Haidara, Ismaeel M Bin-Jaliah, Olaa M Tork, Moshira A Rateb, Hanaa Z Yassin, Zeinb A Al-refaie, Ibrahim M Ibrahim, Samy M Elawa, Laila A Rashed, Noha A Afifi

**Affiliations:** 1 Physiology Department, College of Medicine, King Khalid University, Saudi Arabia; 2 Department of Clinical Biochemistry (Vascular Prevention Clinic), Royal Free Hospital campus, University College of London, UK; 3 Physiology Department, Faculty of Medicine, Cairo University, Egypt; 4 College of Health and Sciences, Kuwait; 5 BiochemistryDepartment, Faculty of Medicine, Cairo University, Egypt; 6 Histology Department, Faculty of Medicine, CairoUniversity, Egypt

**Keywords:** Diabetes, vitamin E, Wistar rats, diabetic cardiomyopathy, apoptosis, oxidative stress, cardiac enzymes

## Abstract

**Aim:**

To investigate the possible role of oxidative stress as a common mediator of apoptosis and cardiac damage in diabetes.

**Materials and Methods:**

This experimental work was conducted on 5 groups of Wistar rats. Group I was the control group. Diabetes type 1 was induced in other groups (by streptozotocin) and animals received insulin or vitamin E (300 mg /kg body weight), both insulin and vitamin E, or no treatment for 4 weeks according to their group. At the end of the study, serum and cardiac tissues were examined for biochemical parameters of cardiac function, oxidative stress and apoptosis. Electron microscopy pictures of cardiac tissue were also evaluated for signs of cardiac damage

**Results:**

Markers of oxidative stress, apoptosis, inflammation as well as manifestations of cardiac damage as assessed by electron microscopy were significantly decreased in rats treated with both insulin and vitamin E when compared with untreated diabetic rats or rats treated with either insulin or vitamin E alone

**Conclusion:**

Administration of both vitamin E and insulin was effective in reducing markers of oxidative stress and apoptosis and improving parameters of cardiac function in experiments animals. Antioxidants might prove beneficial as an adjuvant treatment in addition to insulin in type 1 diabetes associated with manifestations of cardiac complications

## INTRODUCTION

Diabetes represents a serious risk factor for the development of cardiovascular complications such as coronary heart disease, peripheral arterial disease, hypertension, stroke, cardiomyopathy and nephropathy [1]. Identifying risk factors that may lead to diabetes type 2, such as metabolic syndrome (MetS) provides the base for life modification and/or pharmacological intervention in order to prevent cardiovascular complications******[2]. Among other factors, increased oxidative stress has been implicated as a possible mechanism for such complications [3].

In diabetes the circulating free radicals may contribute to progression of heart disease and possibly mediate the process of apoptosis [4], a state where increased oxidative stress is documented [5]. Recent reports provide evidence that high ambient glucose can promote apoptosis *in vitro*, suggesting potential cellular damage as a result of hyperglycemia in diabetes [6]. Though oxidative stress-induced apoptosis was postulated to occur in cases of myocardial infarction [7] it is uncertain whether apoptosis occurs in cardiac muscle during the course of diabetes. Levrand *et al.* [8] postulated that Peroxynitrite (ONOO^−^) triggers apoptosis in cardiomyocytes *in vitro* and in the myocardium *in vivo*. ONOO^−^ is a strong biological oxidant and nitrating species formed from the near-diffusion-limited reaction of the free radicals nitric oxide and superoxide anion [9]. It has been documented that ONOO^−^ formation represents a major mechanism of myocardial injury in various cardiac pathologies including myocardial infarction, chronic heart failure and cardiomyopathy associated with diabetes [10].

ONOO^−^ may cause myocardial cytotoxicity through direct oxidative damage to lipids, proteins and DNA [11], activation of metalloproteinases [12], and the nitration of tyrosine residues within proteins [13]. ONOO^−^ acts as a potent signaling molecule in cardiomyocytes, activating all members of the MAP kinase family [14], and inhibiting the activation of the transcription factor nuclear factor kappa B [15]. One major pathway of ONOO^−^ dependent myocardial cytotoxicity relies on oxidative DNA damage and activation of the nuclear enzyme poly(ADP-ribose) polymerase (PARP), which consumes cellular nicotinamide dinucleotide (NAD) and adenosine triphosphate (ATP), leading to cell necrosis [16].

However, Levrand *et al.* [8] showed that ONOO^−^ exerts potent proapoptotic effects in cardiomyocytes *in vitro* and in the myocardium *in vivo*, characterized by the activation of caspase-3 and the cleavage of PARP. The authors added that ONOO^−^ may represent a major effector of cardiomyocyte apoptosis that may cause myocardial damage and dysfunction in several cardiac pathologies.

On another level, antioxidant administration has been reported to show beneficial effects on parameters of oxidative stress and cardiovascular functions in experimental diabetes [17].

Haidara *et al*. [18] showed that administration of antioxidants, vitamin E or C, may potentially ameliorate endothelial dysfunction and reduce thrombotic tendency in rats with streptozotocin (STZ)-induced DM associated with hypertension.

The aim of the present study was to assess the possible contribution of apoptosis as a mechanism for oxidative stress-induced injury in the myocardium in STZ-induced diabetic rats. We also evaluated the effects of administration of insulin and/or the antioxidant vitamin E on biochemical parameters of oxidative stress and apoptosis, as well as on the histological manifestations of damage in cardiac muscle.

## MATERIALS AND METHODS

The experiments were conducted in the Faculty of Medicine, Cairo University.

### Experimental Animals

50 male albino rats, weighing 170-200 g were used. They were kept in the animal house of Kasr Al-Aini Faculty of Medicine, Cairo University. The rats had free access to standard rat chow and water. They were kept at 22 ± 1ºC temperature at 12 h dark-light cycles.

Rats were randomly divided into 5 groups (each, n=10). Control group (Group 1) received an intraperitoneal (i.p.) injection of 0.1 mol/L sodium citrate buffer (pH 4.5). All other groups (Groups II, III, IV and V) received a single i.p. injection of STZ, 65 mgKg^-1^ body weight [19], freshly dissolved in 0.1 M citrate buffer (pH 4.5). Diabetes mellitus was verified by measuring blood glucose levels (after overnight fast) with the use of glucose oxidase reagent strips (Lif3 scan, Milpitas, CA, USA). Rats having blood glucose level ≥ 300 mg/ dL were considered diabetic [20].

Group II diabetic rats received no treatment during the course of the study, while group III animals (Group III, INS) received 1 unit of insulin, injected subcutaneously (s.c.) every day for 4 weeks. Group IV (E300): diabetic group receiving vitamin E 300 mgKg^-1^ body weight intramuscularly (i.m.) 3 times per week for 4 weeks [21]. Group V (I±E300): Diabetic rats received insulin (1 U), s.c., daily, and vitamin E im 3 times a week for 4 weeks.

The study period lasted for 4 weeks, a period which has been proved to induce detectable diabetic complications in kidneys, skeletal muscles, heart and retina [22].

At the end of 4 weeks, retro-orbital blood samples were obtained under anesthesia, using 40 mgKg^-1^ body weight sodium thiopentone i.p. after an overnight fast. Samples were allowed to clot for 20 min and then centrifuged at 14000 rpm for 10 min for serum separation which was kept at -80 ºC until time of assay of cardiac enzymes. Samples from the left ventricles were removed and prepared for detection of malondialdehyde (MDA), glutathione peroxidase(GPX),cyclic guanosine monophosphate **(**cGMP), gene expression of annexin V, induced nitric oxide synthase (iNOS) and electron microscopic studies of cardiac tissues.

### Chemicals

STZ (Trade name Zanosar) was purchased from Sigma chemical company, St. Louis Missouri, USA, in the form of 1 g vials. The drug was dissolved in 0.1 M sodium citrate (pH adjusted to 4.5). Insulin (Act rapid HM) was purchased from Nordisk Company, in the form of ampoules 100 IU /ml. Vitamin E was purchased from Pharco Pharmaceutical Company in the form of ampoules 250 mg dissolved in arachis oil.

### Measurements

#### Biochemical Parameters

##### 1- Detection of Annexin V and iNOS Gene Expression

About 30 mg of each heart tissue was homogenized in RNA lysis buffer which contains mercaptoethanol then centrifuged at 14000 rpm for 10 min. The supernatant was frozen at -80ºC until examined for gene expression of annexin V and iNOS by RT-PCR.

###### RNA Extraction:

RNA was extracted from heart homogenate using SV-total RNA isolation system kit (Promega, Madison, USA) according to manufacturer’s recommendation and the extracted RNA was measured spectrophotometrically at 280 nm.

###### Reverse Transcriptase and Polymerase Chain Reaction (RT-PCR):

####### cDNA was prepared from RNA as follows:

About 20µg of mRNA was heated at 70 ºC for 5 min with 50 pmol of reverse primer of selected gene (annexin V, iNOS) before adding 5 XRT buffer (50 mM Tris CL, pH8.3. 10 mM dNTPS and 200 units of murine leukemia virus reverse transcriptase in a final volume up 36µL) .RT reaction was carried for 2 h at 37 ºC.

###### Polymerase Chain Reaction (PCR)

5 µL of cDNA was subjected to PCR under the conditions specified below; PCR reaction was carried by adding 50 pmol of each of forward and reverse primer specific to each gene as detailed later.

10 mM dNTPS, 2-5 unit TACL,PCR 10x buffer (containing 100 mM Tris HCL pH 8.3 , KCL 10 mM to final volume 50 µL):

**Table:** 

Primer Sequence	Cycling Condition
1- Annexin V	94 °C 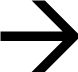 1 min
Sense: 5^-^ GTC TCC ACC CAC TTA	60 °C 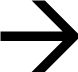 1 min
GTC TAA GTT-3^-^	72 °C 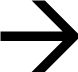 1 min
Anti sense:	Extension
5^-^CCC TGC CAA TGA ACG CTG	72 °C 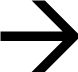 1 min
CCA-3^-^	
2- iNOS	94 °C 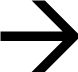 30 sec
Sense: 5^-^GTG AGG ATG AAA ACA	57 °C 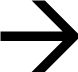 45 sec
TGG- 3^-^	72 °C 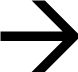 45 sec
Antisense	Extension
5^-^ACC TGC AGG	72 °C 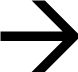 8 min
TTG GAC CA- 3^-^	

###### Agarose Gel Electrophoresis

The amplified PCR product of selected gene were electrophoresed on 1.5 % Agarose gel and were UV visualized after staining with ethidium bromide. UV illuminated gel were photographed. A densitometry system using a Standard DNA of known concentration gene Gel, documentation system was used for analysis (Syngene, Cambridge, UK), Figs. (**1** &amp; **2**).

##### 2) Measurement of MDA

MDA was measured in cardiac tissue homogenate after precipitation of protein by the addition of trichloracetic acid (TCA) then thiobarbituric acid (TBA). TBA reacted with MDA to form thiobarbituric acid product which was measured at 532 nm according [23]. The level of peroxidation was expressed as the amount of MDA in nmol/mg protein.

##### 3) Glutathione Peroxidase (GPX)

GPX was assessed in tissue homogenate using a method based on GSH oxidation by cymene hydroxyl catalyzed by GSH-Px activity (using Wak-Cheme, cat. No: Wak-FR-GPX 80. Germany) according to the manufacturer’s instructions. Absorbance was measured at 340 nm [24].

##### 4) Cyclic Guanosine Monophosphate (cGMP) Assay

Frozen samples stored in 0.1 normal HCL were grounded with a stainless steel mortar, then homogenized and centrifuged at 600g, at 4 ºC for 10 min. The supernatant was used for the cGMP assay by ELISA kit (R&amp;D System, Minnepolis, MN, USA) according to manufacturer’s recommendation [25].

##### 5) Serum Cardiac Enzymes

Creatine kinase (CK) activity was determined using a kit provided by Randox [26].

#### Ultrastructural Studies

Specimens from the ventricles of all animals were processed according to the method of Jans and de-jong [27]. Semi-thin sections “1 µm thickness” were cut by 2 KB ultra-microtome and stained with 1 % toluidine blue for observation. Ultra-thin sections “60-100 nm” thickness were prepared and stained with uranyl acetate and lead citrate to be examined under JOEL EM 1005 transmission electron microscope using an accelerated voltage of 60 KV.

This research was funded and approved by the ethical committee of Kasr Al-Aini Faculty of Medicine, Cairo University.

#### Statistical Analysis

The results are presented as mean ± SD. Comparison were made using paired and unpaired t test one-way ANOVA as required. When a significant F was obtained, multiple comparison post tests were used to determine which groups were significantly different. P ≤ 0.05 was considered significant.

## RESULTS

Table(**1**) shows the effects of diabetes on biochemical parameters of cardiac injury. There is significant increase of serum CK activity in the untreated diabetic group II (P <  0.001) compared with controls. Treatment with either vitamin E or insulin alone significantly decreased the activity of this enzyme compared with the untreated diabetic group (P <  0.05). Treatment with vitamin E and insulin decreased this enzyme back to control levels.

Table(**2**) shows that MDA, an oxidative stress marker, was significantly elevated (P <  0.01) in the diabetic untreated group compared with controls. MDA significantly decreased in insulin treated rats when compared with the untreated group (P<  0.01). Treatment with vitamin E and or insulin decreased MDA significantly below levels in the untreated diabetic group and back to control levels

On the other hand, GPX, which assesses the antioxidant state in tissues, was significantly decreased in untreated diabetic groups compared with controls. Treatment with vitamin E and insulin increased GPX back to control levels.

The antioxidant status in cardiac tissues is better indicated by the ratio of GPX/MDA. This ratio was decreased in the diabetic untreated group and the group only receiving insulin. However, the ratio was increased to the control value in groups treated with either vitamin E alone or vitamin E with insulin

Table (**3**) shows that annexin V levels were significantly elevated (P <  0.001) in the untreated diabetic (group II) cardiac tissues compared with the controls. However, levels were decreased significantly (P <  0.001) in diabetic rats receiving insulin (group III) when compared with untreated diabetic rats. Diabetic animals treated with both insulin and vitamin E showed significant decrease of annexin V in comparison with the untreated diabetic group (P <  0.01) and diabetic rats receiving only vitamin E (P <  0.01) .

Table (**4**) shows the effects of diabetes on the expression of the pro-inflammatory parameters and iNOS. There was a significant increase in both iNOS and cGMP in cardiac tissues of the untreated diabetic group (P <  0.001). Neither insulin alone, nor vitamin E decreased them to control values; however; in the group treated with both insulin and vitamin E, values returned to control levels

### Ultrastructural Electron Microscopic Examination

**Fig. (4):**
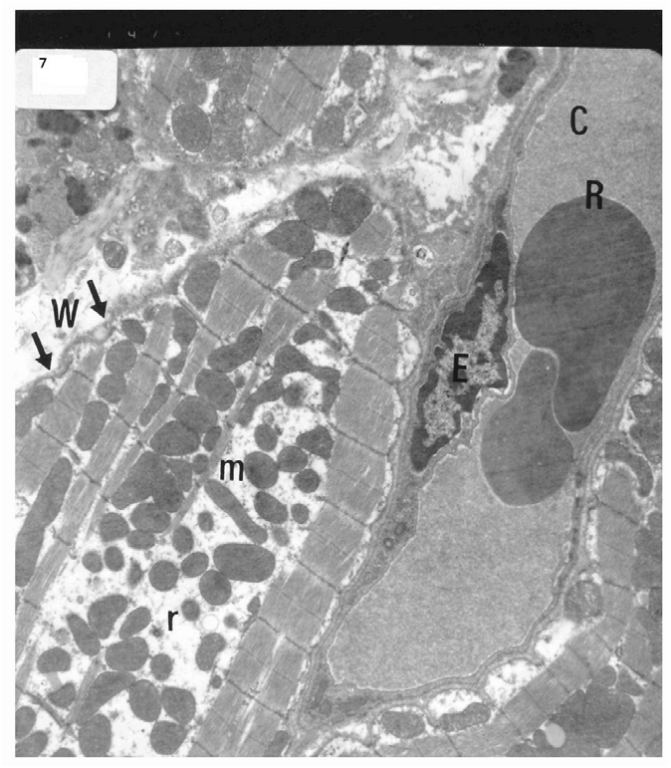
Electron micrograph of an ultrathin section in the ventricular muscle of group II (diabetic rats) showing that mitochondria with dense matrix (m) appear irregularly dispersed. Myofibrils appear widely separated with rarefied cytoplasm of cardiac myocyte (r). Note widening of the intercellular space (W). A blood capillary (C) in the intercellular space shows an endothelial cell (E) in its wall and RBCs in its lumen (R). (Original mag. X 5000).

**Fig. (5):**
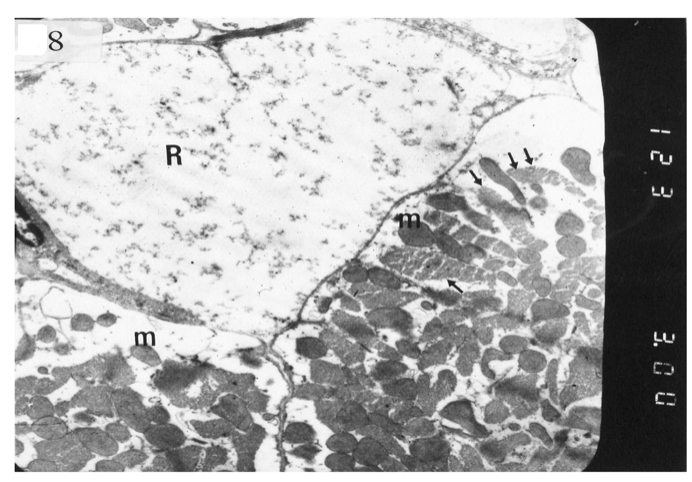
Electron micrograph of an ultrathin section in the ventricular muscle of group II (diabetic rats) showing one rarefied cardiac myocyte (R) with marked myofibrillar lysis. Another myocyte shows rarefied myofibrils (arrows) and mitochondria with dense matrix (m) (Original mag. X 3000).

**Fig. (6):**
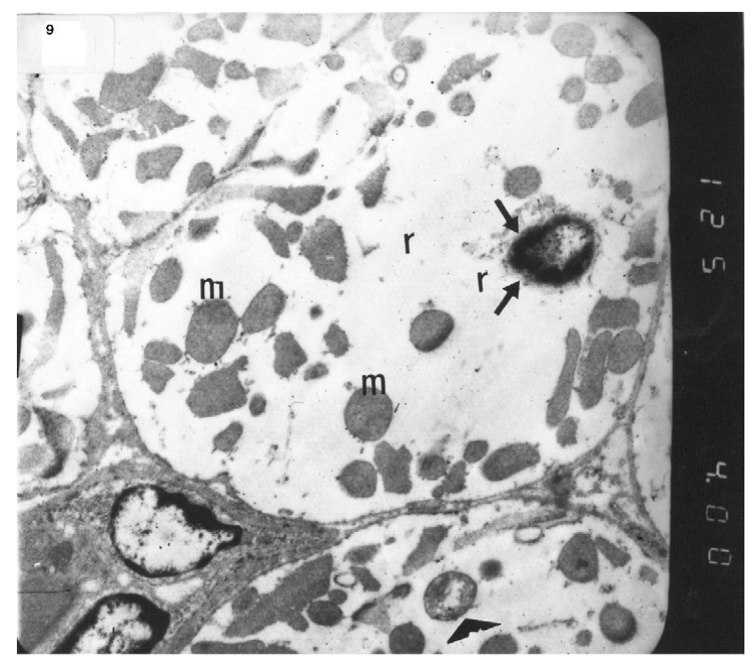
Electron micrograph of an ultrathin section in the ventricular muscle of group II (diabetic rats) showing margination of the nuclear chromatin of a nucleus of a cardiac myocyte (arrows). Myo-fibrils appear completely rarefied (r). Most mitochondria show dense matrix (m) while others show disrupted cristea (arrowhead) (Original mag. X 4000).

**Fig. (7):**
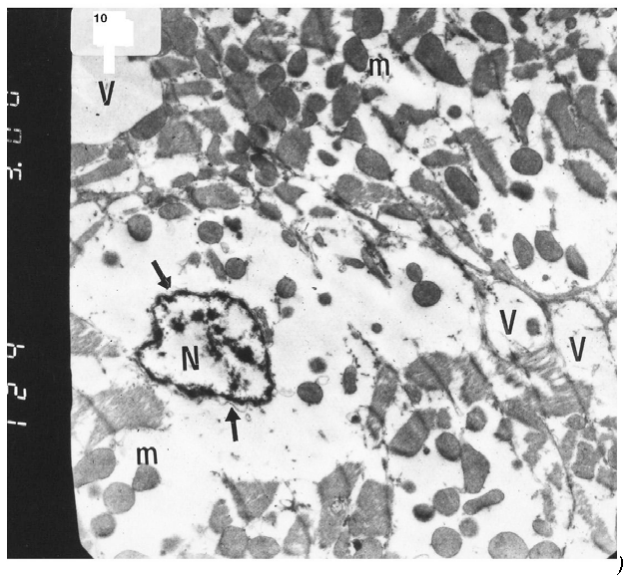
Electron micrograph of an ultrathin section in the ventricular muscle of group II (diabetic rats) showing that the nucleus of a cardiac myocyte (N) has irregular nuclear outline (arrows) with margination of its chromatin. Cardiac myocytes are rarefied with abundant vacuoles (V) and dispersed mitochondria with dense ma-trix (m) (Original mag. X 3000).

Electron microscopic examination of ultra-thin sections of the left ventricular myocardium of the control group (group I) revealed normal histological structure of cardiac myocytes Fig. (**3**). Ventricular sections obtained from untreated rats (group II) showed myofibrillar lysis in the form marked degeneration, disruption and rarefaction of the myofibrils (Figs. **4**, **5**). Nuclei of cardiac myocytes exhibited several degenerative changes where some nuclei showed peripherally condensed marginated chromatin (Fig. **6**). In addition several cardiac myocytes exhibited marked cytoplasmic vacuolations (Fig. **7**).

**Fig. (8):**
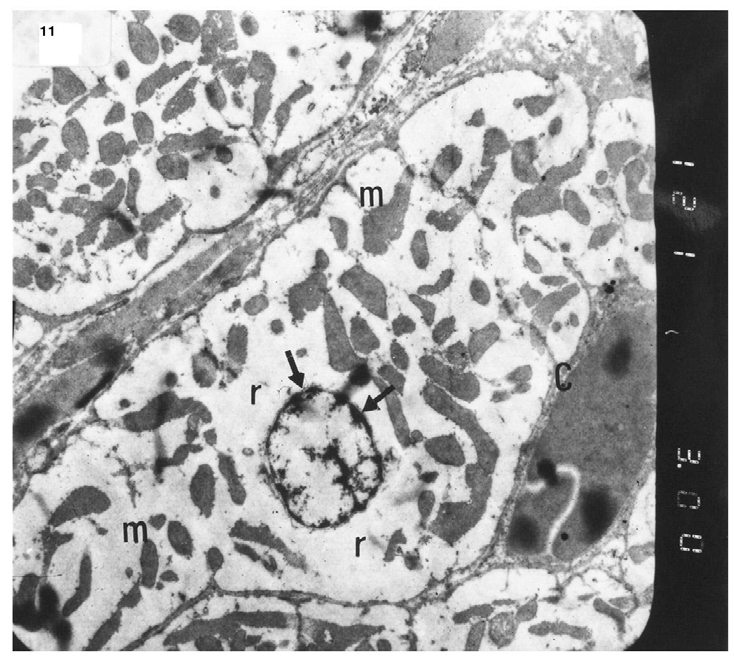
). Electron micrograph of an ultrathin section in the ventricular muscle of group III (diabetic rats receiving insulin) showing margination of nuclear chromatin of a cardiac myocyte (arrows). Myofibrils are rarefied (r). Mitochondria (m) are irregularly dis-persed (Original mag. X 3000).

**Fig. (9):**
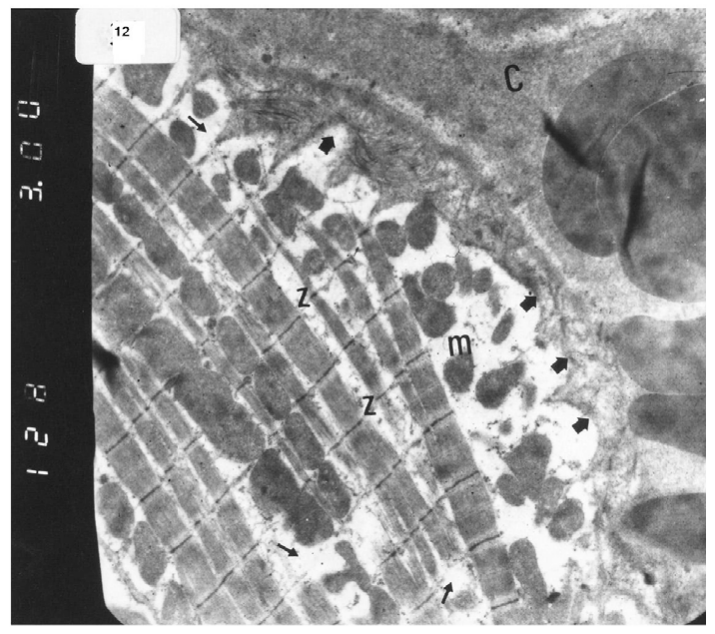
Electron micrograph of an ultrathin section in the ventricular muscle of group IV (diabetic rats receiving vitamin E) showing that most of the myofibrils appear to be forming regular striations yet some of them appear interrupted in certain areas (arrows). Most myofibrils show clear Z lines (Z). Mitochondria (m) with dense matrix are detected between the myofibrils. Sarcolemma appears irregular (short arrows). A blood capillary (C) is seen (Original mag. X 3000).

The ultrastructure of ventricular sections obtained from group III (diabetic rats which received insulin) revealed rarefaction of myofibrils with wide dispersion of mitochondria. Nuclei exhibited condensation of nuclear chromatin (Fig. **8**). The diabetic group which received vitamin E 300 mg showed that most of the myofibrils formed regular striations with clear Z-lines yet striations appeared interrupted in some sites. Mitochondria with dense matrix substance were present between the myofibrils. Sarcolemma appeared markedly irregular (Fig. **9**).

**Fig. (10):**
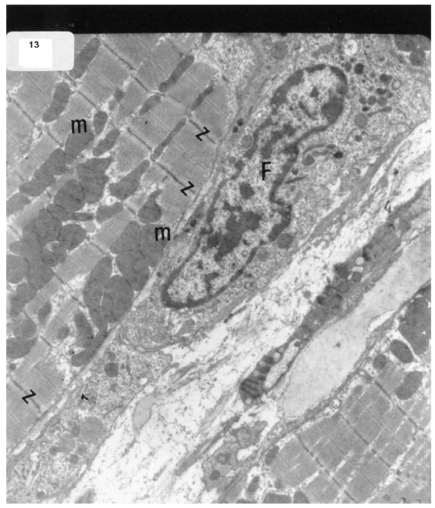
Electron micrograph of an ultrathin section in the ventricular muscle of group V (diabetic rats receiving insulin and vitamin E) showing more regular striations of myofibrils with clear Z lines (Z). Rows of mitochondria (m) are longitudinally arranged between the myofibrils. A fibroblast (F) can be observed in the intercellular space between the cardiac myocytes (Original mag. X 5000).

The ultrastructure of ventricular ultrathin sections of the diabetic group which received both insulin and vitamin E 300 mg showed more regular striations of myofibrils with clear Z-lines. Sarcomeres could be easily detected. Rows of mitochondria appeared longitudinally arranged between the myofibrils (Fig. **10**).

## DISCUSSION

Patients with diabetes type 1 or 2, have a 2-8 fold increased risk of developing cardiovascular diseases such as myocardial infarction, congestive heart failure, cerebrovascular and peripheral arterial diseases [28]. There is growing evidence that oxidative stress associated with diabetes mellitus may promote endothelial dysfunction, hypertension [29], thromboembolism and cardiomyopathy [30]. In both clinical and experimental models of diabetes, reactive oxygen species (ROS)-induced oxidation is considered to be a key factor in causing cardiac injury [31].

High glucose has been postulated to generate ROS and nitrogen species in numerous cell types. Generation of superoxide by high glucose is well described and arises principally *via* the mitochondrial electron transport chain. [32]. Another source of glucose-induced oxidative stress is *via* the polyol pathway where glucose is reduced to sorbitol by aldose reductase in a process that consumes NADPH. This will impair the NADPH-dependent generation of glutathione, an essential cellular antioxidant [33]

Increased ROS generation increases the activity of nuclear factor kappa-B (NF-_k_B) in various cell types including endothelial [34], mesangial [35], and vascular smooth muscle cells [36]. This process is dependent on protein kinase C (PKC) activation [37,38].

Our study showed that diabetic cardiomyopathy may occur in untreated diabetic rats due to long standing hyperglycemia which was demonstrated by structural and functional changes such as increased CK activity and myocardial damage assessed by electron microscopic studies. Assessment of oxidative stress revealed increased MDA content, decreased GPX and GPX/MDA ratio.

Yoon *et al*. [39] postulated that H_2_O_2_ induces an increase in apoptosis signal regulating kinase-1 which cause down regulation of antiapoptotic Bcl-2, disruption of the mitochondrial membrane potential and activation of caspase cascade [40]. High glucose also causes a 2 fold increase in Bax expression, which induced cytochrome C release which in turn stimulates apoptosis activating factor, caspase 9 and caspase 3 [41].

There is evidence that the incidence of apoptosis increases in heart of patients with diabetes [42] and STZ-induced diabetic rats [43]. In this study, increased apoptosis in diabetic cardiac muscles was evident by the increased levels of annexin V in heart tissues of untreated diabetic rats. Annexin V can be used as an apoptotic marker in the heart [44]. Annexin is mainly located in cardiomyocytes. However, it could be relocated to interstitial tissues in ischemic and failing hearts or it could be externalized and exhibit a pro-apoptotic effect in cardiomyocytes [45]. There is a significant increase of plasma annexin V concentration in patients with acute myocardial infarction which could reflect the severity of myocardial damage [46].

It was found that early apoptosis can be assessed and imaged with annexin V scintigraphy in rats [47,48], based on its ability to identify extracellular phosphatidyl-serine, which arises during apoptosis [49].

Annexin V was originally discovered as an antithrombotic activity *in vivo* and it links apoptosis to thrombosis and haemostasis [50]. It is now accepted that cell surface exposure of phosphatidylserine (PS) is an integral part of the apoptotic process. Once committed to die the cell quickly exposes PS at its surface while maintaining the integrity of the plasma [51,52]. PS on the apoptotic cell is thought to serve primarily for the clearance of the dying cell [53]. Annexin V has a high affinity for these surfaces in the complex environment of the tissue and likely form an antithrombotic shield, which reduces the prothrombotic risk associated with apoptosis [54]

Our results also showed that iNOS protein of the heart is elevated in untreated diabetic rats suggesting that inflammation could play a role in the pathogenesis of diabetic cardiomyopathy in type 1 diabetes. Cheng *et al*. [55] and others [56] also found that the activity of iNOS was 3 fold higher in the heart of diabetic rats relative to controls. In addition, they found, that selective inhibition of iNOS restored cardiovascular response to noradrenalin. Apoptosis has been postulated to be involved in the cardiac damage associated with diabetes, sepsis, and dilated cardiomyopathy [57] which are all associated with an enhanced generation of ONOO^−^ within the myocardium [11]. On the basis of these findings, Levrand *et al.* [8] propose that ONOO^−^ may represent a major oxidant species involved in the process of cardiomyocyte apoptosis in these cardiac diseases. These data imply that ONOO^−^-dependent oxidant stress is instrumental in activating proapoptotic signals (caspase-3 and PARP cleavage). The authors added that PARP cleavage as a consequence of ONOO^−^ generation was secondary to the activation of caspase-3, but additional mechanisms may be implicated as well. PARP can also be cleaved in the nucleus of cardiomyocytes through the action of matrix metalloproteinase 2 (MMP-2) [58], known to be activated by ONOO^−^ [12]. Furthermore, Bojunga *et al*. [59] showed that antioxidative treatment was capable of reversing changes in NO-cGMP system and may therefore be an important option for preventing vascular damage in diabetes mellitus. Haidara *et al*., also [30] found that vitamin E was able to modulate the blood pressure and lipid profile in STZ-induced diabetic rats

Vitamin E is an important non-enzymatic natural lipid-soluble chain breaking antioxidant in tissue, red cells and plasma [60,61]. It protects against lipid peroxidation by acting directly with a variety of oxygen radicals to form a relatively innocuous tocopherol radical [62].

Vitamin E prevents H_2_O_2 _– induced apoptosis in remote non-infarcted myocardial cells with prevention of mitochondrial cytochrome C release and activation of caspase 3 [63]. These findings indicated that antioxidant vitamins reduce myocyte apoptosis mediated *via* inhibition of mitochondrial pathway [21]

Vitamin E also seems to inhibit the release of inflammatory mediators from activated monocytes [64] as well as reducing smooth muscle proliferation and platelet aggregation [65].

The present study demonstrates that administration of insulin and vitamin E to diabetic rats reduces oxidative stress and apoptosis with preservation of cardiac function as assessed by the cardiac enzymes and electron microscopy.

Yoshida *et al*. [66] suggested a similar effect in the eye lens as they found that combined treatment of vitamin E and insulin was useful in preventing the development and progression of diabetic cataract. Economides *et al.* [67] did not recommend the use of high dosage of vitamin E in diabetic patients because of their worsening effect on the endothelial or left ventricular function. The dose of vitamin used in this study was based on previous studies [68,69]. The dose is 3-10 times the current recommended dietary allowance but is within human therapeutic range based on body weight [70]. Our findings suggest that the dose of vitamin E (300 mgKg^-1^ body weight) chosen is both clinically relevant but also pharmacologically sufficient to produce antioxidant effects in the experimental setting [21]

## CONCLUDING REMARKS

Our results demonstrated that STZ-induced diabetes in rats leads to functional and structural changes in the heart which include oxidative stress and apoptosis. The changes were significantly ameliorated by administration of insulin and vitamin E which abrogate oxidative stress and produce a cardioprotective effect. The combination of both forms of treatment decreased CK activity and myocardial damage, thus suggesting a strategy which could reduce cardiovascular complications in diabetes mellitus

## Figures and Tables

**Fig. (1):**
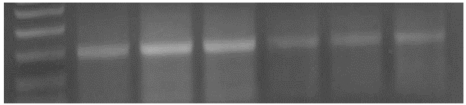
An agarose gel electrophoresis showing products of annexin gene expression; Lane M: PCR marker; Lane 1: gene product in control group. Lane 2&3: gene product in diabetic group. Lane 4: gene product in diabetic group receive insulin. Lane 5: gene product in diabetic group receive vitamin E. Lane 6: gene product in diabetic group receive insulin and vit E

**Fig. (2):**
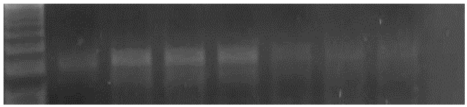
An agarose gel electrophoresis showing product of iNOS gene expression. Lane M: PCR marker. Lane 1: gene product in control group. Lane 2, 3&4: gene product in diabetic group. Lane 5: gene product in diabetic group receive insulin. Lane 6: gene product in diabetic group receive vitamin E. Lane 7: gene product in diabetic group receive insulin and vit E

**Fig. (3):**
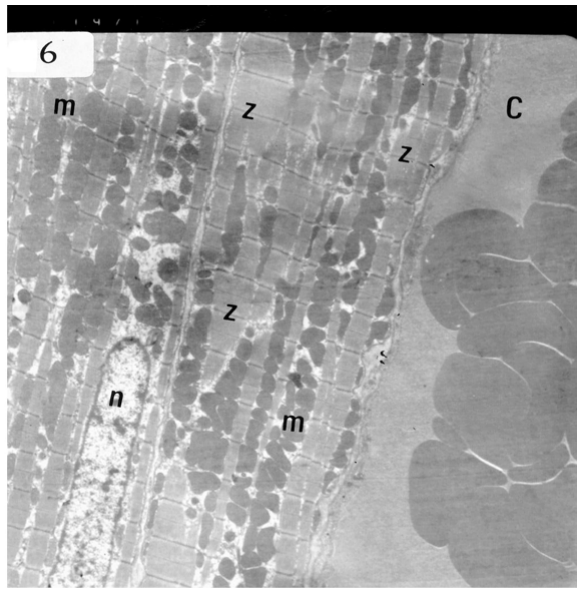
Electron micrograph of an ultrathin section in the ventricular muscle of control rats showing that the nucleus of a cardiac myocyte (n) appears oval with extended euchromatin. Z lines (Z) are easily demarcated. Rows of mitochondria (m) are abundant between the myofibrils. A blood capillary (C) is seen (Original mag. X 3100)

**Table 1:** Creatine Kinase (CK) Activity (U /L Serum) in the Studied Groups 4 Weeks After Induction of Diabetes (n=10 in Each Group)

**Groups**	**Control**	**Diabetic**	**INS**	**E 300**	**I±E300**
**CK**	2.9 ± 0.6	6.0 ± 1.2*	3.4 ± 0.8■	2.8 ± 0.9■	1.9 ± 0.8 ■

Results are mean ±SD; INS= Insulin, E=300: vitamin E 300 mg, I+E=300: insulin 1U sc +Vitamin E 300 mg. CK= Creatine kinase.*Significant with control (P < 0.001); ■ Significant with DM (P < 0.01).

**Table 2:** The Levels of Oxidative Markers MAD (nmol/mg Protein) and the Antioxidant GPX (µ unit/mg Tissue) in the HeartTissues of Studied Groups and Their Ratio GPX/MDA 4 Weeks After Induction of Diabetes (n=10 in Each Group)

**Groups**	**Control**	**Diabetic**	**INS**	**E 300**	**I±E300**
**MDA**	0.1± 0.0	0.3 ±0.1*	0.2± 0.1* ■	0.1 ±0.0■	0.1 ± 0.0■
**GPX**	2.1 ± 0.5	.1 ±0.0*	1.7 ± 0.4■	1.5 ± 0.5■	1.6 ± 0.5■
**GPX/MDA**	19.6± 6.7	0.3± 0.1*	9.0± 3.1*	15.0 ± 7.1■	15.6 ± 3.2■

Results are mean ±SD; INS= Insulin, E=300: vitamin E 300 mg, I+E=300: insulin 1U sc +Vitamin E 300 mg. MDA= malondialdehyde, GPX= glutathione peroxidase.*Significant with control (P < 0.001); ■ Significant with DM (P < 0.01).

**Table 3:** Annexin V Levels (μg /mg Protein) in the Heart Tissues of Studied Groups 4 Weeks After Induction of Diabetes (n=10 in Each Group)

**Groups**	**Control**	**Diabetic**	**INS**	**E 300**	**I±E300**
**Annexin**	403 ± 97	1043 ± 138*	564 ± 108■*	657± 138■*	424 ± 66 ■

Results are mean ±SD;.*Significant with control (P < 0.001); ■ Significant with diabetic (P < 0.01).

**Table 4:** The Levels of iNOS (μg /mg Protein) and cGMP (n mol /mg Protein) in the Heart Tissues of Studied Groups 4 Weeks After Induction of Diabetes (n=10 in Each Group)

**Groups**	**Control**	**Diabetic**	**INS**	**E 300**	**I±E300**
**iNOS**	169 ± 29	451 ± 106*	277 ± 64*■	356 #x00B1; 78*	231 ± 58■
**cGMP **	1.3 ± 0.4	3.6± 0.6*	2.6± 0.5*■	2.3±0.8*■	1.4 ± 0.4■

Results are mean ±SD. Ins= Insulin, E=300: vitamin E 300mg, I+E300: insulin 1U sc +Vitamin E 300 mg, iNOS: inducible nitric oxide synthase, cGMP= cyclic guanosine monophosphate. *Significant with control (P < 0.001);■ Significant with DM (P < 0.01).
